# Regulation of Inflammatory Cytokines for Spinal Cord Injury Repair Through Local Delivery of Therapeutic Agents

**DOI:** 10.1002/advs.201800529

**Published:** 2018-07-31

**Authors:** Hao Ren, Xuri Chen, Mengya Tian, Jing Zhou, Hongwei Ouyang, Zhiyong Zhang

**Affiliations:** ^1^ The Third Affiliated Hospital of Guangzhou Medical University No. 63 Duobao Road Guangzhou 510150 P. R. China; ^2^ Dr. Li Dak Sum & Yip Yio Chin Center for Stem Cell and Regenerative Medicine School of Basic Medical Science Zhejiang University No. 866 Yuhangtang Road Hangzhou 310058 P. R. China; ^3^ Dr. Li Dak Sum & Yip Yio Chin Center for Stem Cell and Regenerative Medicine School of Basic Medical Science Zhejiang University No. 866 Yuhangtang Road Hangzhou 310058 P. R. China; ^4^ Translational Research Center for Regenerative Medicine and 3D Printing Technologies Guangzhou Medical University No. 63 Duobao Road Guangzhou 510150 P. R. China

**Keywords:** inflammatory cytokines, local delivery, spinal cord injuries, therapeutic agents, tissue repair

## Abstract

The balance of inflammation is critical to the repair of spinal cord injury (SCI), which is one of the most devastating traumas in human beings. Inflammatory cytokines, the direct mediators of local inflammation, have differential influences on the repair of the injured spinal cord. Some inflammatory cytokines are demonstrated beneficial to spinal cord repair in SCI models, while some detrimental. Various animal researches have revealed that local delivery of therapeutic agents efficiently regulates inflammatory cytokines and promotes repair from SCI. Quite a few clinical studies have also shown the promotion of repair from SCI through regulation of inflammatory cytokines. However, local delivery of a single agent affects only a part of the inflammatory cytokines that need to be regulated. Meanwhile, different individuals have differential profiles of inflammatory cytokines. Therefore, future studies may aim to develop personalized strategies of locally delivered therapeutic agent cocktails for effective and precise regulation of inflammation, and substantial functional recovery from SCI.

## 1 Introduction

The spinal cord is part of central nervous system (CNS), extending from medulla oblongata to the lumbar region in the vertebral canal. It plays a crucial role in the transmission of motor and sensory information, as well as in the coordination of certain reflexes. The importance of its functions makes SCI one of the most disastrous traumas. SCI is the damage to the spinal cord that results in alterations of its functions, including loss of sensory, motor and visceral functions.^1^ SCI often ends up with paralysis.^2^

The incidence of SCI varies from 3.6 to 195.4 patients per million in different countries worldwide.^3^ For example, in the United States, an estimated 12 500 new cases occur every year, and as many as 276 000 persons are living with SCI. For each patient, the estimated lifetime costs directly associated with SCI range from $1 113 990 to $4 724 181, not including any indirect costs such as loss of wages, fringe benefits and productivity, which average $71 961 per year.^4^ The high prevalence and lifetime costs demonstrate the urgency for therapy development. However, there is currently no effective cure that leads to functional recovery.

After SCI, a series of inflammatory responses are activated. The disruption of the blood‐spinal cord barrier (BSCB) leads to the accumulation of various inflammatory cytokines.^1^ Inflammatory cytokines are a broad category of soluble small proteins that are the mediators and modulators of the complicated functional interactions and reactions of the immune system.^5^ The enormous influence of the inflammatory cytokines on SCI repair has been demonstrated by a large body of research, which will be reviewed here.

Therapeutic agents can be used for the regulation of inflammatory cytokines to promote repair from SCI. However, the fact that the therapeutic agents have difficulty crossing the BSCB restricts the treatments of spinal cord diseases. The BSCB consists of tight junctions and capillary endothelial cells without intracellular fenestrations^6^ and dramatically limits compounds entering the spinal cord from the blood.^7^ There are three main approaches to achieving entry of the agents into the CNS: 1) tailoring therapeutic agents to take advantage of the natural permeability of the BSCB; 2) disruption of the BSCB; and 3) local delivery.^8^ In the field of SCI repair, much research focuses on the utilization of local delivery methods. Therefore, in this review, we will also summarize and discuss the efforts using local delivery of therapeutic agents to regulate inflammatory cytokines and promote SCI repair.

## 2 Roles of Inflammatory Cytokines in SCI Repair

The inflammatory cytokines beneficial and detrimental to SCI repair will be discussed. The criterion by which a certain inflammatory cytokine is determined to be beneficial or detrimental is the direct evidence of functional recovery in vivo. The actions of the beneficial and detrimental inflammatory cytokines are summarized in **Tables** **1** and **2**, respectively.

**Table 1:** Actions of the beneficial inflammatory cytokines

	Cell death blockage	Inflammation regulation	Scar regulation	Neurotrophy	Neurogenesis	Remyelination	Angiogenesis	Pain reduction
EPO	√^9^, ^10^, ^11^, ^12^, ^13^, ^14^, ^15^, ^16^, ^17^, ^18^, ^19^, ^20^, ^21^, ^22^, ^23^, ^24^, ^25^	√^12^, ^17^, ^18^, ^22^, ^26^, ^27^, ^28^	√^15^, ^28^, ^29^	√^30^	√^31^, ^32^, ^33^, ^34^, ^35^	√^15^, ^36^	√^29^, ^30^	
G‐CSF	√^37^, ^38^, ^39^, ^40^	√^41^, ^42^					√^43^	
GM‐CSF	√^44^, ^45^, ^46^		√^47^, ^48^	√^49^	√^49^, ^50^, ^51^, ^52^, ^53^			
IFN‐β	√^54^, ^55^	√^54^	√^56^, ^57^					
IFN‐γ		√^58^, ^59^	√^60^	√^60^				
IL‐4	√^61^, ^62^, ^63^	√^61^, ^62^, ^63^						
IL‐10	√^64^, ^65^, ^66^, ^67^, ^68^, ^69^	√^65^, ^70^, ^71^, ^72^, ^73^						√^64^, ^68^, ^72^, ^74^
IL‐12		√^75^		√^75^	√^75^	√^75^		
IL‐33	√^76^	√^76^	√^76^					
SDF‐1	√^77^	√^77^, ^78^, ^79^, ^80^			√^77^, ^81^, ^82^, ^83^, ^84^		√^77^	

The tick symbol indicates the action that the cytokine has. The numbers correspond to the references.

**Table 2:** Actions of the detrimental inflammatory cytokines

	Cell death	Inflammation	Astrogliosis	Against neurotrophy	Against neurogenesis	Against remyelination	Against angiogenesis	Pain
CXCL10	√^122^, ^123^	√^124^, ^125^			√^122^		√^123^	
IL‐1	√^126^, ^127^, ^128^	√^129^, ^130^, ^131^, ^132^		√^133^				
IL‐1α	√^134^	√^135^						
IL‐1β	√^136^	√^136^	√^136^		√^136^			
IL‐17	√^137^, ^138^	√^137^, ^138^				√^138^		
TNF‐α	√^139^, ^140^, ^141^, ^142^, ^143^, ^144^	√^143^, ^145^			√^146^, ^147^, ^148^	√^148^, ^149^		√^150^

The tick symbol indicates the action that the cytokine has. The numbers correspond to the references.

### 2.1 Inflammatory Cytokines Beneficial to SCI Repair

#### 2.1.1 Erythropoietin (EPO)

EPO, also known as hematopoietin or hemopoietin, was initially discovered as a glycoprotein hormone that controls erythropoiesis.^85^ EPO is also a cytokine whose tissue‐protective activity has been extensively investigated in various injury models,^86^ including SCI. EPO was first reported to dramatically improve functional neurological status in a rabbit ischemia SCI model.^11^ This kind of functional recovery was also demonstrated in rat,^17^, ^87^, ^88^, ^89^, ^90^, ^91^, ^92^, ^93^, ^94^, ^95^, ^96^ mouse,^86^, ^97^ pig,^22^ and other rabbit^98^ models.

One of the most important mechanisms of action might be apoptosis blockade and tissue preservation.^13^ EPO prevented the apoptosis of cells,^12^, ^14^ including neurons^9^, ^10^, ^11^ and oligodendrocytes,^16^ preserved white matter,^15^ and reduced cavitation.^17^ This effect might be because EPO inhibited lipid peroxidation,^20^ reduced caspase‐3^16^ and myeloperoxidase activities,^19^ attenuated the oxidative stress^18^, ^22^ through the nuclear factor erythroid 2‐related factor 2 (Nrf2) signaling pathway,^21^ and increased platelet‐derived growth factor (PDGF)‐B expression.^23^ This tissue protection is possibly related to the downregulation of phospho‐extracellular signal‐regulated kinase (p‐ERK), upregulation of mitogen‐activated protein kinase phosphatase‐1 (MKP‐1)^25^ and phosphorylation of Janus kinase‐2 (JAK2).^24^ The attenuated motor neuron loss might be attributed to recruited CD34^+^ cells and enhanced expression of brain‐derived neurotrophic factor (BDNF).^30^

EPO also limited inflammation,^12^, ^17^ which was thought to be closely related to its anti‐apoptotic effects.^11^ It reduced microglial infiltration,^28^ and levels of TNF‐α,^18^, ^26^ 8‐isoprostane,^22^ thrombospondin‐1 and transforming growth factor‐beta (TGF‐β).^27^

Neuroregeneration is another crucial effect of EPO. EPO decreased phosphacan,^15^ which is an important chondroitin sulfate proteoglycan (CSPG), and reduced astrogliosis^29^ and scar formation,^28^ thus facilitating axonal regeneration.^31^, ^32^ EPO increased nerve growth factor level,^33^ neural progenitor cell (NPC) proliferation,^35^ and synaptogenesis.^34^ It also enhanced remyelination^15^ by promoting oligodendrogenesis.^36^

EPO has other beneficial effects as well, such as promoting angiogenesis and restoration of vascular integrity,^29^ effects that are potentially mediated by vascular endothelial growth factor (VEGF).^30^

#### 2.1.2 Granulocyte Colony‐Stimulating Factor (G‐CSF)

G‐CSF is a 19.6 kDa cytokine^99^ that has been used in clinical applications for the treatment or prevention of chemotherapy‐induced neutropenia, bone‐marrow harvest, and antiinfection treatment.^39^

Urdzíková et al. first found that G‐CSF improved functional recovery after SCI in rats^100^ and concluded that the effect was due to the mobilization of bone marrow stem cells. Later, Yamazaki et al. first applied G‐CSF in a mouse SCI model and reported hindlimb functional recovery.^38^, ^101^ G‐CSF was discovered to attenuate neuronal death^38^ and enhance connectivity.^39^ Moreover, G‐CSF protected oligodendrocytes in SCI repair.^37^ These neuroprotective effects might be attributed to the promotion of angiogenesis^43^ and autophagy^40^ and alternative activation of microglia.^42^ Chen et al. investigated a different delivery method.^41^ Compared to methylprednisolone (MP) administration, direct intrathecal administration of G‐CSF suppressed the expressions of TGF‐β1, CSPGs, and TNF‐α.

#### 2.1.3 Granulocyte‐Macrophage Colony‐Stimulating Factor (GM‐CSF)

GM‐CSF is a 14.2 kDa hematopoietic factor^102^ which is essential for proliferation and differentiation of mature granulocytes and macrophages.^103^

Ha et al. first demonstrated that GM‐CSF improves functional outcome after rat contusive SCI.^46^ The improvement is probably due to the prevention of apoptosis of the cells, including neurons,^46^ via reduction of the expression of the proapoptotic proteins p53, p21, and Bax, and induction of nucleophosmin‐1^44^ and the antiapoptotic protein B‐cell lymphoma 2 (Bcl‐2).^45^ Additionally, GM‐CSF increased BDNF expression by macrophages, and subsequently stimulated axonal regeneration.^49^ Moreover, GM‐CSF suppressed glial scar formation,^47^, ^48^ activated dendritic‐like cells and neural stem cells (NSCs),^53^ and improved the survival of transplanted NSCs.^50^, ^52^ GM‐CSF was also found to improve sensory function by minimizing the abnormal sprouting of sensory nerves.^51^

#### 2.1.4 Interferon‐Beta (IFN‐β)

IFN‐β is one of the type I interferons, which were identified and named because they “interfere” with viral infections. IFN‐β also has antiangiogenic, antiproliferative, immunomodulatory, and cell differentiation activities.^104^, ^105^

IFN‐β was first demonstrated to promote functional recovery in a rat contusive SCI model.^55^ IFN‐β increased heat shock protein 70 levels,^54^ decreased myeloperoxidase activity and lipid peroxidation,^55^ reduced polymorphonuclear leucocyte infiltration, hemorrhage, edema, and necrosis,^54^ and thus preserved tissue structure. IFN‐β also inhibited glial scar formation^57^ by toll‐like receptor 4 signaling.^56^

#### 2.1.5 Interferon‐Gamma (IFN‐γ)

IFN‐γ, one of the type II interferons, has the major activity of immunoregulation. It can be produced by mitogenically or antigenically stimulated lymphocytes.

IFN‐γ was first found to enhance hindlimb function in a mouse contusive SCI model.^60^ IFN‐γ facilitated the secretion of IL‐10 from T helper 1 cells (Th1) and microglia/macrophages,^58^ decreased CSPG expression from reactive astrocytes, and increased the expression of neurotrophic factors, including glial cell line‐derived neurotrophic factor and insulin‐like growth factor‐1.^60^ IFN‐γ is also crucial to the immunological plasticity of the choroid plexus epithelium, allowing the regulated entry of T cells and monocytes, which support CNS repair.^59^

#### 2.1.6 Interleukin‐4 (IL‐4)

IL‐4 is an antiinflammatory cytokine produced by activated T‐lymphocytes.^106^ IL‐4 is associated with the Th2 immune response and the activities of other hematopoietic lineage cells.

IL‐4 was found to regulate acute macrophage activation and confine secondary cavity formation after SCI.^61^ IL‐4 also drove an M2 phenotype, enhanced macrophage recruitment, and reduced tissue damage, finally improving functional outcomes.^62^, ^63^

#### 2.1.7 Interleukin‐10 (IL‐10)

IL‐10 is also an antiinflammatory cytokine. IL‐10 can be synthesized by Th2, monocytes/macrophages, astrocytes, and microglia^107^, ^108^ and suppresses monocyte/macrophage inflammatory responses and the production of multiple cytokines, cell adhesion molecules, reactive oxygen, and nitrogen intermediates.^107^, ^109^, ^110^, ^111^

IL‐10 has been demonstrated to promote functional recovery in SCI models in rats^66^, ^67^, ^68^, ^69^, ^72^, ^73^, ^74^ and mice.^58^, ^64^, ^65^, ^70^, ^71^ IL‐10 affects inflammation through regulation of activation of microglia/macrophages^70^, ^71^ and astrocytes,^72^ as well as reducing the production of TNF‐α,^72^, ^73^ IL‐1β, S100β, and inducible nitric oxide synthase (iNOS).^65^ IL‐10 also increases expression of Bcl‐2 and B‐cell lymphoma‐extra large (Bcl‐xl),^66^ thus promoting neuronal survival,^68^, ^69^ and decreasing cavitation^67^ and tissue loss.^64^, ^65^ Further, IL‐10 limits the onset and severity of injury‐induced pain behaviors.^64^, ^68^, ^72^, ^74^

#### 2.1.8 Interleukin‐12 (IL‐12)

IL‐12 is a 70‐kDa heterodimeric cytokine produced by dendritic cells (DCs), macrophages, monocytes, and B cells.^112^ IL‐12 has the ability to regulate innate and adaptive immune responses, especially the Th1 immune response.

Yaguchi et al. demonstrated in a mouse SCI model that IL‐12, secreted from DCs, promoted functional recovery.^75^ The recovery might be because IL‐12 increased the number of activated microglia/macrophages and DCs, as well as BDNF expression, and subsequently improved neurogenesis and remyelination.

#### 2.1.9 Interleukin‐33 (IL‐33)

IL‐33 belongs to the IL‐1 cytokine family.^113^ IL‐33 can be produced by endothelial cells, epithelial cells and fibroblasts. IL‐33 intracellularly regulates gene expression,^114^, ^115^ and is also an alarm mediator when released from injured cells.^116^, ^117^, ^118^

IL‐33 was shown to reduce secondary injury and improve functional recovery in a mouse contusive SCI model.^76^ IL‐33 reduced TNF‐α expression, cytotoxic TNF‐α^+^/CD4^+^ cells, and M1 polarization, increased M2 polarization and T‐regulatory cells, and induced a shift toward Th2. Thus, IL‐33 decreased demyelination and tissue loss, as well as astrogliosis.

#### 2.1.10 Stromal Cell‐Derived Factor‐1 (SDF‐1)

SDF‐1, also known as CXCL12, is a member of the CXC chemokine subfamily. SDF‐1 has activities in cell migration, proliferation, differentiation and survival,^119^ through receptors, including CXCR4^120^ and CXCR7.^121^

Zendedel et al. first corroborated that SDF‐1 improved functional recovery in a rat contusion model.^77^ SDF‐1 promoted transmigration of monocytes^79^ and macrophages^80^ into the injured cord, boosted astroglia and microglia responses,^77^ reduced the levels of IL‐18, IL‐1β, TNF‐α, and NLRP3,^78^ and thus regulated inflammation. SDF‐1 also decreased apoptosis and enhanced angiogenesis, chemotaxis and proliferation^77^ of cells, including NSCs,^81^, ^82^ subsequently enhancing axonal sprouting.^83^, ^84^

### 2.2 Inflammatory Cytokines Detrimental to SCI Repair

#### 2.2.1 Chemokine (C‐X‐C Motif) Ligand 10 (CXCL10)

CXCL10, also known as interferon‐inducible protein 10 kDa (IP‐10), is a CXC chemokine. CXCL10 inhibits angiogenesis^151^, ^152^, ^153^ and preferentially recruits Th1^154^ through CXCR3. The actions of the detrimental inflammatory cytokines are also summarized in Table 2.

Gonzalez et al. first showed that antibody neutralization of CXCL10 enhanced functional recovery in a mouse dorsal hemisection SCI model.^124^ The neutralization reduced apoptosis,^122^ inhibited T‐lymphocyte invasion^124^ and inflammation,^125^ and thus enhanced tissue sparing.^123^ CXCL10 neutralization also enhanced angiogenesis and the subsequent axon sprouting.

#### 2.2.2 Interleukin‐1 (IL‐1)

IL‐1 is a soluble cytokine produced by various types of cells, including monocytes and macrophages. IL‐1 has the ability to activate T cells and facilitate the host response to infection.^133^ IL‐1 has two distinct forms, IL‐1α and IL‐1β, which exhibit only 26% homology but very similar biologic activities.^155^, ^156^

IL‐1 receptor antagonist (IL‐1ra), a selective endogenous receptor antagonist, blocks the actions of IL‐1α and IL‐1β.^157^, ^158^ Nesic et al. first applied IL‐1ra in a rat contusive SCI model.^128^ Akuzawa et al. first found that IL‐1ra is helpful to recovery of motor function in rabbit ischemic SCI.^127^ IL‐1 knockout was also found to promote locomotor activity in a mouse transection SCI model.^129^ IL‐1ra reduced p38 mitogen‐activated protein kinase^126^ and caspase‐3 activation,^128^ increased BDNF expression^133^ and thus limited apoptosis and necrosis.^127^ Additionally, IL‐1 increased expression of TNF‐α^129^, ^130^ and entry of neutrophil and type I “inflammatory” monocyte,^132^ and activated microglia/macrophages,^129^, ^131^ thus aggravating inflammation.

There are also investigations distinguishing IL‐1α from IL‐1β. Tonai et al. found that injection of exogenous IL‐1α into the spinal cord increased production of cyclooxygenase‐2 and eicosanoid, and enhanced migration of polymorphonuclear leukocytes in a rat compression SCI model.^135^ Bastien et al. found that IL‐1α gene deletion in a mouse model protected oligodendrocytes through the survival factor Tox3, reduced lesion volume and promoted functional recovery.^134^

IL‐1β, a 17‐kDa protein, is one of the most extensively studied proinflammatory cytokines. Liu et al. first demonstrated that IL‐1β impaired locomotion recovery in a rat glutamate‐induced SCI model.^159^ This impairment was also confirmed in a mouse compressive SCI model.^136^ These results may be due to IL‐1β increasing the level of TNF‐α and Bax as well as the activity of caspase‐3, decreasing Bcl‐2 expression and subsequent neuronal survival, exacerbating lesion size and astrogliosis, and dampening axonal plasticity.^136^

#### 2.2.3 Interleukin‐17 (IL‐17)

IL‐17, also known as IL‐25, is mainly produced by T cells and their precursors. IL‐17 is closely related to the origin of a type 2 immune response, which is characterized by the differentiation of Th2 and the production of the type 2 cytokines.^160^, ^161^

Hill et al. first showed that IL‐17 hindered functional recovery in a mouse contusive SCI model.^137^ This might be because IL‐17 recruited B cells, dendritic cells, neutrophils,^137^ and Th cells,^138^ and increased lesion size and demyelination.

#### 2.2.4 Tumor Necrosis Factor‐Alpha (TNF‐α)

TNF‐α is a pleiotropic pro‐inflammatory cytokine that can be produced by activated macrophages as well as other mammalian mononuclear leukocytes.^162^ By two cell surface receptors, TNF receptor 1 and 2, TNF‐α induces diverse biological activities, including necrosis and apoptosis.^163^

TNF‐α has been demonstrated to dampen functional recovery in rat and mouse SCI models.^164^, ^165^ TNF‐α induces AMPAR trafficking and enhances excitotoxicity.^139^, ^140^ Additionally, TNF‐α increases NO production,^143^ elevates neutrophil infiltration and inflammation,^145^ induces edema and microvascular permeability disturbances,^142^ and initiates apoptosis of cells,^140^, ^141^ including neurons and oligodendrocytes.^143^ TNF‐α inhibits the survival and differentiation of oligodendrocyte precursor cells,^149^ as well as the survival of transplanted NSCs,^146^ and thus limits remyelination and neural regeneration.^148^ TNF‐α undermines spinal plasticity via alterations in AMPARs.^147^ TNF‐α‐induced alterations in electrophysiological properties of axons may also contribute to neurological deficits.^144^ Moreover, TNF‐α promotes below‐level neuropathic pain after SCI.^150^

## 3 Local Delivery of Therapeutic Agents Regulates Inflammatory Cytokines and Promotes SCI Repair

**Figure 1:**
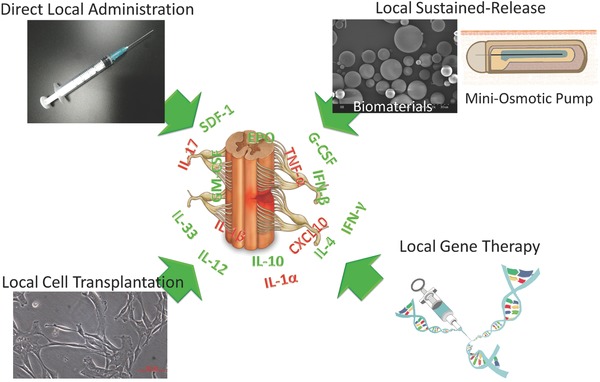
Local delivery of therapeutic agents, including direct administration, sustained‐release, cell transplantation and gene therapy, regulates inflammatory cytokines and promotes SCI repair. Cytokines in green are beneficial to SCI repair, while those in red are detrimental. Arrows indicate the regulation of inflammatory cytokines and promotion of repair.

Local delivery means that the therapeutic agents are administered directly into the lesion site or that an agent administration system is constructed for local application. In comparison with systemic administration, local delivery overcomes the BSCB and thus improves the concentration of the local agent with less damage to other sensitive organs.^166^, ^167^ Here, we classify local delivery into 4 subunits: direct administration, sustained release, cell transplantation, and transgene, and discuss their promotion of SCI recovery through regulation of inflammatory cytokines in animal models (**Figure** **1**).

### 3.1 Direct Local Administration

Administrating agents around or in the lesion site can achieve sufficient therapeutic effects of regulating inflammatory cytokines and promoting injury repair. The directly administered agents include inhibitors, cytokines and other therapeutic agents.

#### 3.1.1 Inhibitors

Several investigations have used antibodies to directly inhibit the levels of certain inflammatory cytokines. Local applications of anti‐TNF‐α antibodies reduced apoptosis,^141^, ^143^ and attenuated BSCB disturbances and cord pathology,^164^ thus enhancing neurorepair.^168^ The suitable combination of antibodies against TNF‐α and NOS also improved neuroprotection and functional recovery.^164^, ^168^ In addition to anti‐TNF‐α antibodies, CCL20‐neutralizing antibodies have been found to promote function recovery as well.^169^ They reduced the levels of IL‐1β, IL‐6, TNF‐α, and IL‐17, and decreased Th17 cell recruitment.

Antagonists are also important inhibitors. Intrathecal injections of IL‐1ra decreased apoptosis after rat SCI.^126^ The antagonists against TNF‐α increased levels of IL‐4 and IL‐10 and decreased the level of IL‐1β as well as tissue damage, improving functional outcome.^170^, ^171^

#### 3.1.2 Cytokines

In the SCI epicenter, directly administered cytokines include EPO, G‐CSF, and VEGF. The intrathecal injection of recombinant human EPO (rhEPO) was demonstrated to enhance the SDF‐1/CXCR4 axis in transplanted bone marrow stromal cells (BMSCs), thus increasing their migration toward the lesion site.^26^ rhEPO also reduced the apoptosis of BMSCs and improved neurological outcome. G‐CSF administered intrathecally had antiinflammatory and anti‐apoptotic effects by directly regulating the activity of MAPK and Akt pathways, suppressing the expression of TGF‐β1 and TNF‐α, and improving neurological function in both ischemic and contusive SCI models.^41^, ^172^ The direct injection of VEGF was also found to regulate inflammatory cytokines post‐SCI.^173^ VEGF decreased the levels of IL‐1β and TNF‐α, reduced motor neuron loss, and improved function.

#### 3.1.3 Other Therapeutic Agents

Moreover, there are other therapeutic agents, including chondroitinase ABC, Annexin A1 (ANXA1) and 11‐dehydrosinulariolide. Multiple lumbar puncture injections of chondroitinase ABC promoted IL‐10 expression and inhibited IL‐12b expression after SCI.^174^ ANXA1 is suggested to be an endogenous neuroprotective agent that mediates the antiinflammatory actions of glucocorticoids.^175^ The intraspinal injection of ANXA1 significantly decreased IL‐1α expression, inhibited the inflammatory response, and reduced tissue damage. The intrathecal injection of the coral‐derived 11‐dehydrosinulariolide was found to attenuate SCI‐induced upregulation of TNF‐α, increase M2 polarization, and improve functional recovery.^176^

### 3.2 Sustained Release

Sustained release forms are designed to release agents at a controllable rate in order to maintain a constant drug concentration for a period of time. To regulate inflammatory cytokines after SCI, sustained agent release can be achieved mainly through two methods: biomaterial systems and pumps.

#### 3.2.1 Biomaterial Systems

In SCI models, the topically applied biomaterial systems involve gelatin sponges, hyaluronan methylcellulose (HAMC), and biomaterial particles.

*Gelatin Sponge*: Gelatin sponges are sterile sponges with porous structure that work primarily as hemostatic agents. They are pH‐neutral and biodegradable and, therefore, also used for local delivery.^177^, ^178^ After SCI, topically implanted gelfoam sponges soaked with IL‐12 increased the number of activated ameboid‐type microglia/macrophages and their BDNF expression, accompanied by increased remyelination and promotion of functional recovery.^75^ In another study, gelfoam sponges loaded with GM‐CSF modulated apoptosis and promoted neuroprotection.^179^ Further, Li et al. developed an NT‐3/fibroin‐coated gelatin sponge scaffold, which could continually release NT‐3 for 28 days.^180^ Its local application decreased TNF‐α level, CD68‐positive cells and cavity formation and improved neural regeneration.

**Figure 2:**
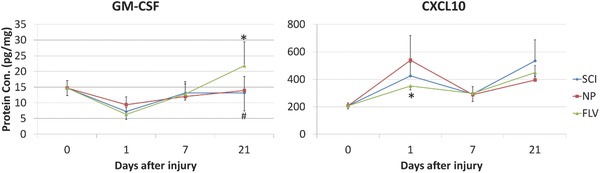
Local delivery of flavopiridol nanoparticles increased GM‐CSF and decreased CXCL10 protein levels in spinal cord extracts in a rat right hemisection model. The right‐hemisection SCI model was established as previously described.^188^ 0, 1, 7, or 21 days after injury, 0.5–1 cm spinal cord tissue at injury site was harvested and weighed before protein extraction. The protein levels were measured using a Procarta Multiplex Cytokine Immunoassay kit (Affymetrix, Fremont, CA). The protein levels were calculated as picogram per milligram spinal cord tissue. SCI, no treatment after injury. NP, local delivery of blank nanoparticles after injury. FLV, local delivery of flavopiridol nanoparticles after injury. *n* = 5. Data are mean ± standard deviation, *p < 0.05 versus NP, ^#^ *p* < 0.05 versus SCI.

*Gelatin Sponge: Particles*: Both microspheres and nanoparticles have been applied in animal models. Transplanting chitosan‐based atorvastatin calcium microspheres into the SCI site attenuated the expression of TNF‐α, IL‐1β and IL‐6, and improved functional outcomes.^181^ Estrogen is known to have anti‐inflammatory and neurotrophic properties.^182^, ^183^, ^184^, ^185^, ^186^ The implantation of estrogen nanoparticles modulated various inflammatory cytokines including IL‐1α, IL‐1β, IL‐10, IL‐12p70, IL‐17a, TNF‐α, MIP‐1α, MCP‐1, and IFN‐γ after SCI.^187^ Flavopiridol is a cell‐cycle inhibitor which facilitates repair from SCI. In our research, local delivery of flavopiridol in poly (lactic‐co‐glycolic acid) nanoparticles significantly decreased TNF‐α, IL‐1β, and IL‐6, as well as elevated IL‐10 expression.^188^ Recently, using a Procarta Multiplex Cytokine Immunoassay kit (Affymetrix, Fremont, CA), we found that flavopiridol nanoparticles also increased GM‐CSF and decreased CXCL10 protein levels in spinal cord extracts in a rat right hemisection model (**Figure** **2**).

*Gelatin Sponge: HAMC*: The University of Toronto developed a new biomaterial called hydrogel of HAMC as a drug carrier for SCI treatment.^189^ Compared with intrathecal or intraperitoneal EPO injection, HAMC EPO delivery had better results in tissue sparing and functional recovery post‐SCI.^190^ Interestingly, HAMC had some therapeutic functions on its own,^189^, ^191^ such as improving the outcome of post‐traumatic syringomyelia.^191^ Intrathecal injection of HAMC reduced IL‐1α levels and lesion volume and finally promoted neurobehavioral recovery from arachnoiditis caused by SCI.

#### 3.2.2 Osmotic Pumps

Osmotic pumps are small, infusion pumps for administrating agents in animals. They are convenient, cost‐effective and capable of maintaining drug levels.^192^ These pumps have been widely employed to release agents for regulation of inflammatory cytokines post‐SCI, including cytokines, inhibitors, and other therapeutic agents.

*Cytokines*: Intrathecal administration of SDF‐1α by osmotic pumps reduced the levels of inflammatory cytokines,^78^ including IL‐18, IL‐1β, and TNF‐α, protected neurons,^77^ and promoted sprouting of corticospinal tract axons and motor recovery.^78^, ^84^

*Inhibitors*: In rat SCI models, EGFR blockers from osmotic pumps depressed IL‐1β and TNF‐α production, inhibited microglia/astrocyte activation and glial scar/cavity formation, and promoted axonal outgrowth with functional recovery.^193^ Central administration of a soluble TNF blocker reduced spinal cord damage and improved locomotor function.^171^ Etanercept, a blocker of TNF‐α, was administered into the SCI site by pumps, attenuated microglial activation and prevented the development of mechanical hypersensitivity.^194^ Local pumping of IL‐1ra decreased IL‐1β,^159^ blocked its combination with its receptor, and completely abolished the increased apoptosis after SCI.^128^

*Other Therapeutic Agents*: Metabotropic glutamate receptor 5 (mGluR5) has neuroprotective properties. mGluR5 agonist treatment through pumps reduced TNF‐α, microglial activation and lesion volume and improved functional recovery.^195^ Agomir‐210, a chemical mimic of microRNA‐210, attenuated the expression of proinflammatory TNF‐α and IL‐1β, upregulated IL‐10, and inhibited apoptosis in SCI models.^196^ Pumping a connexin43 mimic into the injured spinal cord decreased TNF‐α and IL‐1, reduced secondary damage and eventually promoted hindlimb function.^197^

### 3.3 Local Cell Transplantation

Recently, cell transplantation has been one of the hotspots in SCI repair. Transplanted cells can release cytokines/chemokines and influence the inflammatory response.^198^ Various investigations in SCI models have shown that local cell transplantation regulates inflammatory cytokines and facilitates recovery. Donor cells for local transplantation involve stem cells and somatic cells.

#### 3.3.1 Stem Cells

*Mesenchymal Stem Cells (MSCs)*: MSCs are mainly derived from bone marrow and may have the potential ability to reduce the acute inflammatory response. Cells transplanted directly into the lesion site in SCI animal models reduce the expression of TNF‐α,^198^, ^199^, ^200^, ^201^, ^202^, ^203^, ^204^ IL‐1β,^200^, ^201^, ^203^ IL‐6,^198^, ^199^, ^200^, ^202^ IL‐2, IL‐4, IL‐12,^199^ IFN‐α,^200^ IL‐10, TGF‐β1,^201^ MMP‐9,^202^, ^204^ CCL2, CCL5, CCL10,^202^ GM‐CSF, and tissue inhibitor of metalloproteinases,^203^ and increase the expression of IL‐4,^198^, ^201^ IL‐13,^198^ CCL5,^199^ GM‐CSF,^202^ leptin, and ciliary neurotrophic factor.^203^

Compared to direct transplantation, alternative methods could enhance the therapeutic effects of MSCs. MSCs encapsulated with alginate microencapsulation could shift the phenotype of macrophages from M1 to M2 shown by evaluation of the expression of CD206 and IL‐10, attenuate the expression of IL‐1β, IP‐10, and macrophage inflammatory protein 1‐α (MIP1‐α), and increase IL‐6 expression, thus promoting tissue repair.^205^ Implantation of peptide‐modified inverted colloidal crystal scaffolds with BMSCs downregulated glial fibrillary acidic protein (GFAP) and TNF‐α expression, and enhanced neuronal survival.^206^ Further, canine MSCs overexpressing heme oxygenase‐1 (HO‐1) promoted functional recovery through anti‐inflammatory and neuronal regeneration effects.^207^ Inflammatory markers such as IL‐6, cyclooxygenase‐2 (COX‐2), phosphorylated‐signal transducer, and activator of transcription 3 (p‐STAT3), and galactosylceramidase (GALC) showed decreased expression in experimental dogs. Additionally, local transplantation of MSCs with plumbagin alleviated SCI through downregulation of NF‐κB, p65 and TNF‐α.^208^ Moreover, the combined therapy of MSC local transplantation and hyperbaric oxygen (HBO) provided MSCs with a beneficial microenvironment for survival and thus promoted the functional recovery of SCI rats.^200^

*Neural Stem Cells (NSCs)*: NSCs are self‐renewing cells that can generate neurons, oligodendrocytes, and astrocytes.^209^, ^210^, ^211^ Cheng et al. found that NSC transplantation into the SCI epicenter could reduce neutrophils, regulate macrophage activation, attenuate mRNA levels of inflammatory cytokines such as TNF‐α, IL‐1β, IL‐6, and IL‐12, and improve functional recovery.^212^ In addition, stem cells combined with other therapeutic systems may be more effective in curing SCI. For example, Wu et al. developed a eukaryotic expression plasmid for expressing human EPO, transfected NSCs, and then injected the cells into the subarachnoid cavity.^88^ These EPO‐NSCs promoted motor function and SCI repair. Kim et al. produced a hypoxia‐inducible GM‐CSF‐expressing plasmid and transfected NSCs.^213^ Transplantation of these NSCs increased GM‐CSF, enhanced NSC survival and neuronal differentiation, and promoted functional recovery. Since TNF‐α contributed to the neural cell death, the combination therapy of TNF‐α inhibitor and NSCs could effectively protect transplanted NSCs, promote remyelination and neural regeneration, and improve locomotor function.^146^

*Dental Stem Cells*: Dental stem cells include dental pulp stem cells (DPSCs) and dental follicle stem cells (DFSCs), representing another possible way to treat SCI. Both DPSCs and DFSCs significantly inhibited expression of IL‐1β to reduce inflammation damage,^214^ and DPSCs were neuroprotective via inhibition of TNF‐α overexpression.^215^

*Blood Stem Cells*: Takahashi et al. used G‐CSF to mobilize peripheral blood stem cells (PBSCs) from peripheral blood, and PBSC intraspinal transplantation promoted angiogenesis and serotonergic fiber sparing, preserved myelin, and improved function recovery.^216^ A mononuclear cell layer of human umbilical cord blood stem cells injected into the contusion site downregulated the expression of TNF‐α, TNFR1, and TNFR2 and thus inhibited neuronal apoptosis.^217^

#### 3.3.2 Somatic Cells

*Olfactory Ensheathing Cells (OECs)*: OECs replace necrotic or apoptotic neural cells and help remyelination and neurotrophin secretion.^218^, ^219^ Transplanted OECs increased the reactivity of GFAP, tomato lectin, IL‐1β, and iNOS in the lesioned cord of SCI rats at 7 days, which were all reduced at 14 days post‐injury.^220^ While Schwann cells (SCs) promote axonal regeneration by secreting basement membrane components type IV collagen and laminin,^221^ OEC and SC cotransplantation reduced astrocyte/microglia/macrophage infiltration and inhibited proinflammatory factor (IL‐6 and TNF‐α) secretion, increased the levels of anti‐inflammatory factors (IL‐10 and IL‐13), repaired cystic cavities, and improved functional recovery.^222^

*Other Somatic Cells*: Skin‐coincubated macrophages injected into the caudal border of the lesion elevated the secretion of IL‐1β and BDNF, reduced the secretion of TNF‐α and showed meaningful recovery in rats.^223^ In addition, transplantation of Wharton's jelly cells (WJCs), which are isolated from umbilical cord Wharton's jelly tissue, inhibited IL‐1β expression, promoted nerve growth factor (NGF) expression in spinal cord tissues, and improved neurological function recovery.^224^

### 3.4 Local Gene Therapy

Gene therapy refers to the techniques and strategies to genetically transform or modify cells for healing or attenuating disease conditions. Various animal experiments have shown that regulation of inflammatory cytokines and repair from SCI is facilitated by local gene therapy, including liposome‐mediated and virus‐mediated gene therapy.

#### 3.4.1 Liposome‐Mediated Gene Therapy

Ito et al. built a liposome‐mediated IFN‐β gene delivery system and applied it locally after SCI. This gene therapy inhibited glial scar formation and promoted functional recovery by deactivating the MEK‐ERK pathway.^57^ Liposomes were also used to deliver miR‐199b.^225^ Intrathecal delivery of miR‐199b reversed the upregulation of IL‐1β and TNF‐α, and attenuated acute SCI.

#### 3.4.2 Virus‐Mediated Gene Therapy

Viral vector injections are also used for expressing specific proteins in targeted areas.^226^ To regulate inflammatory cytokines post‐SCI, herpes simplex virus (HSV), lentivirus, adenovirus and poliovirus have been locally applied in animal experiments.

HSV is neurotropic, lives without replication, has a large genome and is easily operated in tissue culture.^227^ HSV was used as a vector to express IL‐10 and EPO locally in SCI mice. Local injection of IL‐10 vector was discovered to promote survival of neurons.^66^ Wang et al. made an HSV vector coding EPO and injected it locally after SCI. Their study showed an enhanced tissue sparing, preserved axons and promotion of synaptogenesis, in accordance with a diminution of the injury size and a significant functional improvement.^34^

Lentivirus is the most widely used virus in gene therapy studies aimed at inflammatory cytokines after SCI. Local applications of lentivirus‐mediated regulation of both TNF‐α^228^ and IL‐10^71^ promoted functional recovery. In addition to inflammatory cytokines, local lentivirus‐mediated regulation of other proteins also affected inflammation and facilitated neurorepair. Ji et al. edited a lentivirus BDNF‐overexpressing vector and injected it into the SCI epicenter.^229^ The injection resulted in reduced IL‐1β and TNF‐α, elevated IL‐10 and IL‐13, and promotion of M2 polarization. This prevented CST retraction and improved motor function. Local lentivirus‐mediated RNA interference (RNAi) of the regulator of calcineurin 1 suppressed the increase in IL‐1β and TNF‐α and improved behavioral performance in injured rats.^230^ Local transfection of siRNA against the TNF‐like weak inducer of apoptosis decreased IL‐1β and TNF‐α, and enhanced locomotor functional recovery in injured mice.^231^

Adenovirus is also widely applied for local inflammatory cytokines in SCI. Local adenovirus‐mediated RNAi of IL‐1β decreased TNF‐α and neuronal loss, and improved locomotor function.^232^ Adenovirus‐mediated local overexpression of BMP and activin membrane‐bound inhibitor decreased IL‐1β, IL‐6, and TGF‐β, and facilitated functional recovery.^233^

Poliovirus vectors have the capacity for self‐amplification and for expressing foreign proteins without infecting other cells. Jackson et al. intrathecally injected poliovirus vectors to express IL‐10 in SCI animals.^70^ This treatment inhibited microglia activation and improved functional recovery.

## 4 Clinical Studies

**Figure 3:**
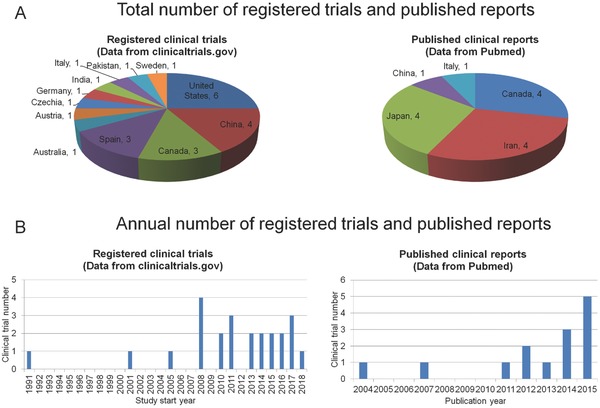
The literature reviews of clinical use of local agent delivery to regulate inflammatory cytokines and treat SCI, using the search engines of both clinicaltrials.gov for the registry of clinical trials, and PubMed for the published clinical reports.

Quite a few promising clinical studies have tested different treatments for SCI.^234^ As inflammatory cytokines play important roles in SCI, there are some studies focusing on inflammatory cytokines for SCI therapy. We found 24 registered clinical trials (clinicaltrials.gov) and 14 published clinical reports on inflammatory cytokines in SCI. The United States and China have the most registered clinical trials, with 6 and 4 trials, respectively. Canada, Iran and Japan have the most published clinical reports. Each of the 3 countries has 4 reports (**Figure** **3**A). There is a rising trend in the number of registered clinical trials and published clinical reports, with 62.5% of the registered trials and 85.7% of the published reports coming after 2010, showing the current interest for the potential of regulation of inflammatory cytokines for SCI healing (Figure 3B).

Here, we summarized and discussed all of the relevant clinical studies of SCI repair through regulation of inflammatory cytokines (**Table** **3**). According to the method of agent administration, we divide these trials into four types: subcutaneous injection, intravenous injection, intramuscular injection and local delivery.

**Table 3:** Clinical studies regulating inflammatory cytokines for treatment of SCI

Delivery method	Agent	Clinical outcome	Year	Ref.
Subcutaneous injection	5 µg kg^−1^ G‐CSF per day for 5 days	Safe	2013	235
	5 µg kg^−1^ G‐CSF per day for 7 days	Improved ASIA motor score, light touch and pinprick sensory scores	2014	236
	100 µg day^−1^ EPO for 3 days	Pain relieved and lower limbs strength recovered the next day; ambulation after 1 year	2015	237
	75 IU kg^−1^ rhEPO three times a week for 6 weeks	Ulcer status improved	2004	238
	75 IU kg^−1^ rhEPO three times a week for 6 weeks	Negative	2015	239
Intravenous injection	5 or 10 µg kg^−1^ day^−1^ G‐CSF for 5 days	No side effects. Neurological improvements in both motor and sensory functions	2012	240, 241
	10 µg kg^−1^ day^−1^ G‐CSF for 5 days	Muscle strength of upper and lower extremities improved	2014	242
	10 µg kg^−1^ day^−1^ G‐CSF for 5 days	Higher ASIA motor scores and less severe side effects, when compared with MPSS	2015	243
	1500 IU kg^−1^ EPO over 30 min	Improved motor function and shorter recovery time, including functional class and ambulation	2007	244
	500 UI kg^−1^ EPO at 24 and 48 h	ASIA Impairment Scale improved when compared with MP	2015	245
	Combination of EPO and MPSS. MPSS 30 mg kg^−1^ initially and 5.4 mg kg^−1^ h till 23 h if admitted within 3 h and till 47 h if recruited within 3–6 h after injury; 500 IU mL^−1^ rhEPO immediately and 24 h later	ASIA scores dramatically increased, when compared with MPSS only	2014	246
	Combination of EPO and MP. 500 IU mL^−1^ rhEPO immediately and 24 h later; MP 30 mg kg^−1^ initially and 5.4 mg kg^−1^ h^−1^ for 23 h	Negative	2015	247
Intramuscular injection	Combination of EPO and MP. 1000 IU kg^−1^ EPO intramuscularly three times per week for 8 weeks; MP 30 mg kg^−1^ initially and 5.4 mg kg^−1^ h^−1^ for 23 h	AISA score and activity of daily living improved	2011	248

### 4.1 Subcutaneous Injection

Subcutaneous injection is a shot given into the subcutis. This form of administration is used to give small amounts of certain kinds of medicine. At present, clinical trials with subcutaneous injections of inflammatory cytokines, including G‐CSF and EPO, have shown promise.

#### 4.1.1 G‐CSF

Derakhshanrad et al. recruited 19 patients, all of whom received G‐CSF (5 µg kg^−1^ day^−1^) subcutaneously for 5 days.^235^ Some patients showed mild side effects (rash, fever, bone pain, neuropathic pain, and spasticity) that disappeared in a week. The results demonstrated that subcutaneous injection of G‐CSF is safe for SCI patients. After a year, this team recruited more patients. After receiving the identical G‐CSF administration for 7 consecutive days, patients with motor‐incomplete SCI showed better improvement, including improved American Spinal Cord Injury Association (ASIA) motor score and light touch and pinprick sensory scores.^236^

#### 4.1.2 EPO

In another case, a 42‐year‐old woman showed low response to routine drug treatment after cervical discectomy that directly led to SCI.^237^ Then, a derivative of EPO was injected subcutaneously (100 µg day^−1^) for 3 days as an addition to her medications. The next day, her pain was relieved, and lower limb strength recovered gradually. After one year, she walked without help and returned to her previous job.

Additionally, rhEPO administration has had a positive impact on SCI patients with pressure ulcers (PUs) associated with Anemia of Chronic Disease (ACD).^238^, ^239^ Four patients received rhEPO (75 IU kg^−1^) subcutaneously three times a week for 6 weeks.^238^ After the treatment, ulcer status was improved in all patients, as shown by the reduction in ulcer depth, decrease in the wound surface areas and improvement in wound appearance and damage degree. However, in another trial, rhEPO administration at a dose of 75 IU kg^−1^ was not enough to significantly improve patient prognosis.^239^

### 4.2 Intravenous Injection

Intravenous injection is a method that infuses liquid substances directly into the vein, through which they are then carried by blood circulation and brought into effect systemically. Currently, intravenously administered agents being examined in clinical trials include G‐CSF and EPO.

#### 4.2.1 G‐CSF

In 2012, Sakuma et al. reported that G‐CSF administration was safe and had neuroprotective effects.^240^ A total of 5 patients received G‐CSF (5 µg kg^−1^ day^−1^) intravenously for 5 days, while another 10 patients received 10 µg kg^−1^ day^−1^. None of the 15 patients showed side effects, and treatment resulted in neurological improvements in both motor and sensory functions. Along with the above research, this team also obtained similar results in patients with acute SCI caused by falls and road trauma, as well as athletic injury.^241^

In consideration of the influence of different injury sites, this group continued a further trial only on patients with cervical injury.^242^ The ASIA motor scores collected at one week, three months and one year after primary injury were dramatically increased in the G‐CSF group compared to those in the control group, suggesting that intravenous injection of G‐CSF improved the muscle strength of the upper and lower extremities.

In addition, compared with high‐dose methylprednisolone sodium succinate (MPSS) treatment, intravenous administration of G‐CSF produced significantly higher ASIA motor scores.^243^ Furthermore, less severe side effects such as pneumonia were observed in patients treated with G‐CSF, indicating that intravenous administration of G‐CSF is safer and more effective.

#### 4.2.2 EPO

In clinical studies, EPO has also been administered intravenously. Ten paraparetic patients with malignant extradural spinal cord compression (MESCC) received 1500 IU kg^−1^ EPO by chemotherapy infusion pump over 30 min and showed improved motor function and shorter recovery time, including functional class and ambulation.^244^ Additionally, Costa et al. found that intravenous administration of EPO resulted in a meaningful clinical improvement in ASIA Impairment Scale scores compared with MP treatment.^245^ Meanwhile, the combination of EPO and MP has been confirmed in clinical trials to be beneficial for SCI treatment.^246^ ASIA scores were dramatically higher in patients treated with the combination than in those treated with MPSS only.

However, in another trial, administration of rhEPO together with MP did not improve the functional outcome of patients with traumatic cervical SCI, which might be due to insufficient dosage (500 IU kg^−1^) of rhEPO, expression of the EPO receptor or the route of administration.^247^

### 4.3 Intramuscular Injection

Intramuscular injection is the injection of a substance directly into a muscle where medicines are absorbed quickly by abundant blood vessels. Intramuscular injection of EPO is also used in SCI treatment.

Xiong et al. recruited 63 patients with spinal cord ischemia‐reperfusion (I‐R) injury and found that combined administration of 1000 IU kg^−1^ EPO injected intramuscularly and MP injected intravenously markedly improved AISA score and the activity of daily living compared to MP administration alone.^248^

### 4.4 Local Delivery

Two methods of local delivery have been applied in clinical trials of SCI: intrathecal delivery and intraspinal injection. These trials have been registered (clinicaltrials.gov), but currently they have no posted results.

Intrathecal delivery is a very important means of local administration and is relatively convenient as it can be done by lumbar puncture. BMSCs, adipose‐derived MSCs and umbilical cord‐derived MSCs have been utilized respectively in the intrathecal delivery of 6 trials (NCT02481440, NCT01186679, NCT03308565, NCT01393977, NCT02482194, and NCT02570932). The outcome measures include TNF‐α, TGF‐β, IL‐1β, IL‐6, iNOS, IL‐10, subsets of T‐lymphocytes, and C‐reactive protein.

There are two clinical trials using intraspinal injection (NCT01046786 and NCT02917291). The investigators at the Chinese University of Hong Kong and The University of Hong Kong slowly injected cord blood mononuclear cells into the posterior grey matter after laminectomy and opening of the dura. In the other trial, the intramedullary injection of allogeneic adipose‐derived MSCs was used.

## 5 Future Directions

Current studies demonstrate that regulation of inflammatory cytokines plays a significant role in SCI repair both in animal models and clinical trials. Nevertheless, two problems exist: enabling the efficacy of agents at small dosages and reducing side effects. Local delivery is showing outstanding advantages in solving these problems. Local delivery of therapeutic agents and personalized therapy have great potential in the future.

### 5.1 Local Delivery Enables the Efficacy of Agents at Small Dosages

We have learned that subcutaneous or intravenous injections of inflammatory cytokines are widely used for the clinical treatment of SCI. As mentioned above, EPO administration of 1500^244^ and 1000 IU kg^−1^ 248 can improve functional recovery in SCI patients, while it fails to produce apparent therapeutic effects in a dose of 500 IU kg^−1^.^247^ Thus, we propose that local delivery of a small‐dose administration may have a better effect, and we hope to apply these local delivery methods to clinical studies in the future.

Moreover, a large number of animal experiments have revealed that local delivery of therapeutic agents can regulate inflammatory cytokines and promote functional recovery, which further illustrates that local agent delivery can promote SCI repair in the clinical setting. Local, but not systemic administration of TNF inhibitor, was therapeutic for traumatic SCI in mice.^171^ Central administration of XPro1595 reduced damage to the lesions and improved locomotor function, while peripheral administration of anti‐TNF therapies was inefficient.

At the same time, SCI could occur in any spinal cord site, so local delivery is more accurate and economic than systemic administration.

### 5.2 Local Delivery Reduces Side Effects

In systemic agent delivery, high doses of agents produce side effects. In clinical trials of SCI, the restriction of the blood–brain barrier and short half‐life of IL‐10 required a high dose of IL‐10 in systemic delivery,^249^ which resulted in increased susceptibility to Klebsiella pneumonia,^250^ Listeria monocytogenes,^251^ and Streptococcus pneumonia infections.^252^ In human beings, systemic administration of MP also induced side effects, including wound infection, hyperglycemia, severe pneumonia, and delayed wound healing.^253^ Importantly, local delivery indeed reduced the MP‐induced side effects in animal experiments.^254^ Further, the emergence of local delivery methods in more and more clinical trials (NCT02481440, NCT01186679, NCT03308565, NCT01393977, NCT02482194, NCT02570932, NCT01046786, and NCT02917291) is also showing their great potential. Thus, enabling efficacy at small dosages and reducing side effects, local delivery of therapeutic agents is very promising for the future.

### 5.3 Future Personalized Strategies of Locally Delivered Therapeutic Agent Cocktails

Local delivery of a single agent only affects a small portion of the inflammatory cytokines, and different individuals have differential profiles of inflammatory cytokines. Huang et al. found that inflammatory cytokine profiles were related to monocyte (MO) phenotypes in SCI patients.^255^ According to the expression of CD14 and CD16, the inflammatory MO subpopulation contained two subgroups: M1‐dominant subpopulations (CD14^low^/CD16^+^) and M2‐dominant subpopulations (CD14^high^/CD16^+^). They found that the M1‐dominant subpopulation showed higher levels of IL‐12 and CXCL10 in the plasma, while the M2‐dominant subpopulation presented with more IL‐10, IL‐15, and IL‐7. Therefore, future studies may aim to develop personalized strategies for locally delivered therapeutic agent cocktails for effective and precise regulation of inflammation and substantial functional recovery from SCI.

## Conflict of Interest

The authors declare no conflict of interest.
